# A New Heterogeneous Catalyst Obtained via Supramolecular Decoration of Graphene with a Pd^2+^ Azamacrocyclic Complex

**DOI:** 10.3390/molecules24152714

**Published:** 2019-07-26

**Authors:** Matteo Savastano, Paloma Arranz-Mascarós, Maria Paz Clares, Rafael Cuesta, Maria Luz Godino-Salido, Lluis Guijarro, Maria Dolores Gutiérrez-Valero, Mario Inclán, Antonio Bianchi, Enrique García-España, Rafael López-Garzón

**Affiliations:** 1 Department of Chemistry “Ugo Schiff”, University of Florence, Via della Lastruccia, 3-13, 50019 Sesto Fiorentino, Italy; 2 Department of Inorganic and Organic Chemistry, University of Jaén, 23071 Jaén, Spain; 3 Institute of Molecular Sciences, University of Valencia, Calle José Beltrán Martínez, 2, 46980 Paterna (Valencia), Spain

**Keywords:** catalysis, Sonogashira, cross coupling, palladium catalyst, azamacrocycles, surface adsorption, supramolecular interactions

## Abstract

A new G-(H_2_L)-Pd heterogeneous catalyst has been prepared via a self-assembly process consisting in the spontaneous adsorption, in water at room temperature, of a macrocyclic H_2_L ligand on graphene (G) (G + H_2_L = G-(H_2_L)), followed by decoration of the macrocycle with Pd^2+^ ions (G-(H_2_L) + Pd^2+^ = G-(H_2_L)-Pd) under the same mild conditions. This supramolecular approach is a sustainable (green) procedure that preserves the special characteristics of graphene and furnishes an efficient catalyst for the Cu-free Sonogashira cross coupling reaction between iodobenzene and phenylacetylene. Indeed, G-(H_2_L)-Pd shows an excellent conversion (90%) of reactants into diphenylacetylene under mild conditions (50 °C, water, aerobic atmosphere, 14 h). The catalyst proved to be reusable for at least four cycles, although decreasing yields down to 50% were observed.

## 1. Introduction

The formation of carbon-carbon bonds is of central importance in different areas of chemistry and, above all, it is prerequisite for all forms of life on earth. Transition metals have a special ability to activate various organic compounds and, as a result, they may be able to catalyze the formation of new bonds. One metal that was used early for the catalytic formation of C-based bonds was palladium. Indeed, the palladium catalysts have given access, through the formation of C-C, C-N and C-O bonds, to new products that otherwise would have required far more complicated procedures [1].

While many early processes employed homogeneous catalysts [1,2], the research has recently focused on the construction of designed heterogeneous Pd-catalysts as they offer more practical solutions, in terms of easy of recovery, reuse and economical convenience compared to expensive homogeneous catalysts, which, moreover, remain as impurities in the final reaction mixtures. 

**Figure 1:**
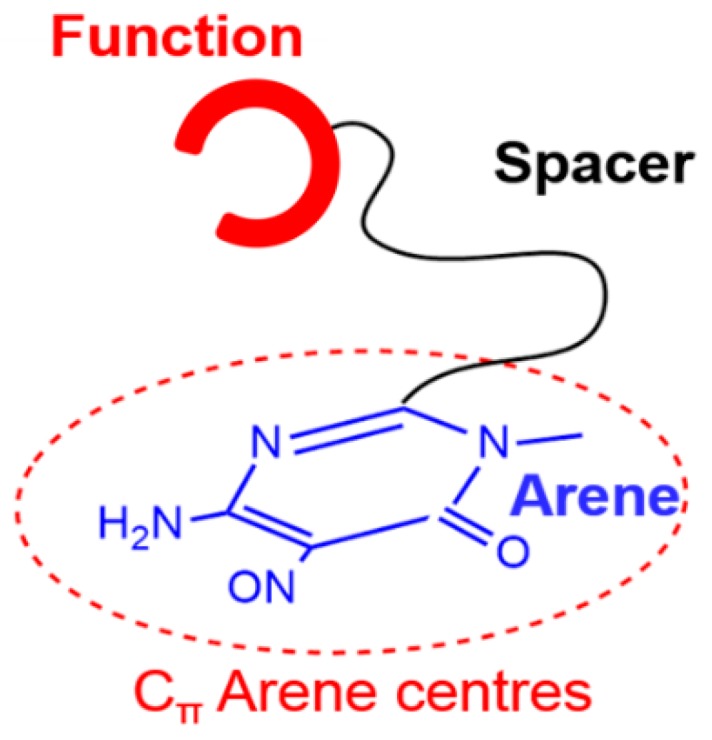
General structure of an Ar-S-F ligand.

Different types of materials have been proposed as possible solid supports, including many carbon-based materials [3,4,5,6,7,8,9]. In this respect, some of us have recently shown that the 6-amino-3,4-dihydro-3-methyl-5-nitroso-4-oxopyrimidine group can be profitably exploited for the non-covalent functionalization of carbon materials thanks to its ability to π-π stack irreversibly with arene centers [10]. Following this strategy, different ligands, herewith represented as Ar-S-F (Figure 1), were appended on activated carbon (AC) [11,12,13,14,15,16,17,18] and on multi-walled carbon nanotubes (MWCNT) [19,20,21] to obtain hybrid materials that were employed for the recovery of both metal cations [12,13,14,15,16,21] and anions [17,18] from aqueous media and for the preparation of Pd-catalysts with high efficiency towards oxygen reduction reactions in alkaline media [19], Cu-free Sonogashira cross coupling reactions [20] and hydrogenation processes [11].

The involvement of supramolecular forces (mostly π-π stacking) and of the hydrophobic effect for the attachment of similar ligands onto the surface of carbon materials has considerable advantage with respect to the covalent functionalization, because the adsorption of the Ar-S-F molecules takes place through a self-assembling process at room temperature that allows preserving the structure of the carbon substrate and maintaining a strict control of the functionalization of the graphitic surface, relative to the amount of catalytic sites and the homogeneity of their distribution. Furthermore, the hydrophilic character of the F function favors the dispersion of CNTs in water.

**Figure 2:**
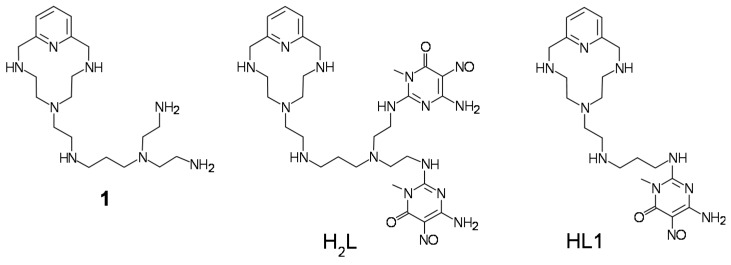
The ligand H_2_L studied in this work, its precursor **1** and the previous HL1 analogue.

In a recent paper [20] we reported the preparation of the Ar-S-F type HL1 ligand based on 6-amino-3,4-dihydro-3-methyl-5-nitroso-4-oxopyrimidine (Figure 2), of the MWCNT-(HL1) hybrid material and of its Pd^2+^ derivative MWCNT-(HL1)-Pd (with Pd^2+^ coordinated into the macrocyclic ring) and we showed that the latter behaves as a very efficient catalyst in the copper-free Sonogashira cross coupling reaction between iodobenzene and phenylacetylene during, at least, four reaction cycles. Nevertheless, a slight constant deactivation was observed along these reaction cycles which was proved to be due to some lixiviation of the (HL1)-Pd complexes from the MWCNT surface under reuse.

In an attempt to prevent similar lixiviation phenomena, we designed and synthesized a modification of HL1 containing two pyrimidine anchorages, instead of one and we implanted this new H_2_L ligand (Figure 2) on graphene (G) believing that the flat surface of G would favor a stronger interaction with the flat pyrimidine units of H_2_L compared to the curved plane of the MWCNTs. 

The special 2D structure endows graphene with unique physical and chemical properties [22,23] that make it an excellent material for catalytic applications [24]. For example, graphene that has a large number of edges or defects can be directly used as a catalyst [25,26] but also modified graphene has been developed through both covalent and supramolecular functionalization to obtain metal-based catalysts [3,4,27,28].

Following the procedure previously adopted for the preparation of MWCNT-(HL1)-Pd [20], the new G-(H_2_L)-Pd catalyst studied in the present work was constructed via a spontaneous supramolecular self-assembly process, taking place in water at room temperature, consisting in the non-covalent attachment of H_2_L onto G, to form the G-(H_2_L) hybrid material which is successively decorated with Pd^2+^ ions inside the macrocyclic unit. Based on previous experience [19,20], the macrocyclic unit of H_2_L is well suited to form a thermodynamically very stable and kinetically inert Pd^2+^ complex, preserving a coordination site on the metal ion available for the catalytic activity. 

The copper-free Sonogashira coupling was the test reaction used to compare the catalytic properties of G-(H_2_L)-Pd and MWCNT-(HL1)-Pd. Although Cu(I) plays an important role in the Sonogashira reaction, in recent years there has been a growing commitment to the development of procedures that work in the absence of Cu(I) [29,30] to avoid the drawbacks connected to the presence of this metal ion, such as the induction of a Glaser type oxidative homocoupling of the terminal alkyne to yield the corresponding diene [31] and inhibitory effects [32,33]. Phosphine-palladium catalysts, as well as palladium complexes with polydentate ligands bearing nitrogen and oxygen donors have been extensively used to perform copper-free Sonogashira reactions under homogeneous conditions [34] but the air sensitivity of phosphine ligands, demanding strict anaerobic conditions and the difficulty of recovering/recycling such homogeneous catalysts has hampered their application on a large scale. Conversely, our G-(H_2_L)-Pd catalyst can be prepared under mild conditions (water, aerobic, room temperature) and employed in the copper-free Sonogashira reaction between iodobenzene and phenylacetylene under mild conditions (water, aerobic, 50 °C). Furthermore, the catalyst can be recovered, by means of a common filtration and reused. 

We describe here the results of this study.

## 2. Results and Discussion

### 2.1. Acid-Base Properties of H_2_L

**Figure 3:**
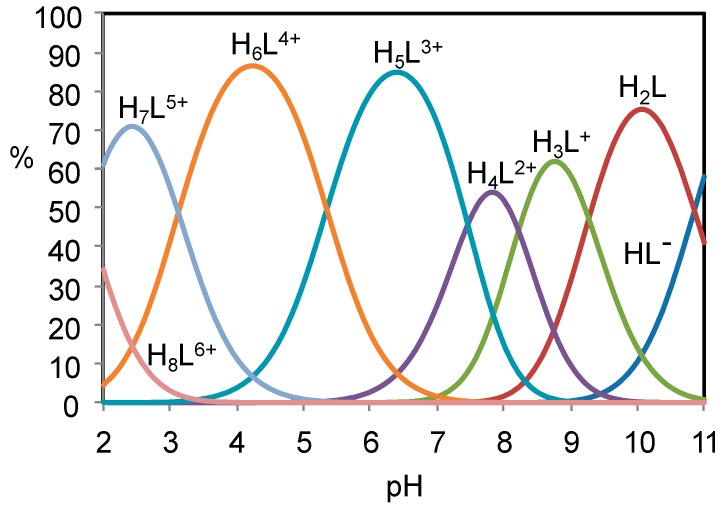
Distribution diagram of the protonated species formed by H_2_L. [H_2_L] = 1 × 10^−3^ M.

The protonation/deprotonation equilibria involving H_2_L in the pH range 2.5–11 were studied by means of potentiometric (pH-metric) titrations in 0.15 M NaCl aqueous solution at 298.1 K. Analysis of the titration curves showed that, in the pH range 2.5–9.5, H_2_L has six basic groups undergoing protonation, while it is involved in a deprotonation equilibrium in the pH range 9.5–11. The equilibrium constants determined for these processes are reported in Table 1 in the form of protonation constants. A distribution diagram of the different species formed by H_2_L as a function of pH is reported in Figure 3.

As previously observed for analogous polyamines bearing the same pyrimidine residue, ligand deprotonation occurring at high pH (corresponding to protonation log*K* = 10.85) is due to the amine groups directly connected to the pyrimidine ring. H_2_L contains two similar groups but only the deprotonation of one of them has been observed in the investigated pH range. Nevertheless, the short name H_2_L adopted for the ligand accounts for the presence of two deprotonable groups. On the other side, protonation occurring in the most acidic region involves the pyrimidine nitroso groups. 

**Figure 4:**
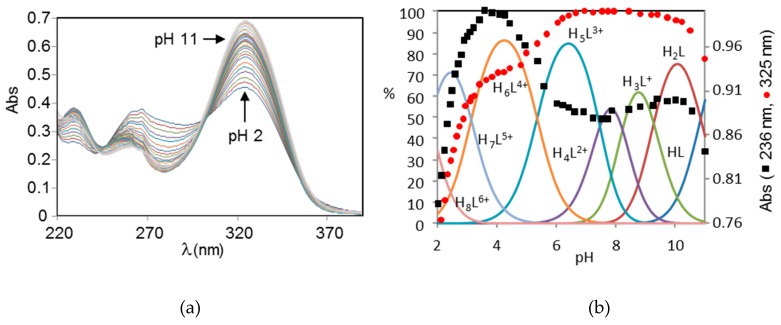
(**a**) UV spectra of H_2_L at different pH values. (**b**) Variation with pH of the 236 nm and 325 nm normalized absorbances of H_2_L superimposed to the distribution diagrams of the species formed.

This assignment of the protonation sites is confirmed by the variation with pH of the ligand adsorption spectra. As shown in Figure 4 the near-UV spectra of H_2_L display three bands at about 230, 260 and 325 nm corresponding to allowed π/π* transitions between π-orbitals of the pyrimidinic group overlapping with the band at about 260 nm of the pyridinic one. The spectra display a marked pH dependence in acidic and alkaline solution, when protonation involves the pyrimidine chromophore. Nevertheless, the variation of the 325 nm absorbance is affected by a slowdown around pH 4, while the band at 236 nm returns to lower values from this pH on. This behavior is likely due to the involvement of the pyrimidine chromophore in the formation of intramolecular hydrogen bonds. In the pH region 7–10, the spectra are almost invariant since protonation takes place on the non-chromogenic aliphatic amine groups.

### 2.2. Formation of Pd(II) Complexes in Solution

Complexation of Pd^2+^ by H_2_L is a very slow process. Once the complex has formed, however, the protonation equilibria involving the complex at pH above 2.5 can be studied, without kinetic complications, until pH 8.5 when precipitation of the complex occurs. Accordingly, we managed to determine the complex protonation constants (Table 1), by using the same potentiometric method employed to study ligand protonation (see above) in 0.15 M NaCl aqueous solution at 298.1 K. A distribution diagram of the complex species formed as a function of pH is reported in Figure S1.

In the case of the (HL1)-Pd complex previously studied, it was found that the ligand uses only three nitrogen atoms (pyridine and secondary nitrogen atoms) of its macrocyclic unit to bind Pd^2+^ which completes its square planar geometry by coordination of a chloride anion from the medium [20]. This binding mode is also verified for the Pd^2+^ complex with H_2_L. In fact, ion chromatography analysis of a solution containing equimolar quantities of ligand and [PdCl_4_]^2−^, equilibrated at pH 4 and 298 K until completeness of the complexation reaction (the UV-Vis spectrum of the mixtures showed no variations after four days), showed that the concentration of free Cl^-^ was three times that of the original [PdCl_4_]^2−^, that is, one Cl^-^ anion remained coordinated to Pd^2+^. 

The UV spectra of complex solutions (Figure S2) showed that, in the acidic region, protonation corresponding to the lowest protonation constant (Table 1) occurs on the pyrimidine group, while deprotonation of the amine group directly connected to the pyrimidine ring takes place above pH 10, as already observed for the metal-free ligand. Unfortunately, it was not possible to determine the equilibrium constant related to this deprotonation process due to the above cited complex precipitation occurring over pH 8.5 under the concentration conditions used for potentiometric measurements.

### 2.3. Preparation and Characterization of the G-(H_2_L) Hybrid

A detailed analysis of the properties of the supporting material (G) employed in this work can be found in a previous paper [35]. After washing with distilled water, G (1–3 graphene layers as constituent units) was used without further treatment; C (94.8%) and O (4.6%) are the main constituents according to XPS analysis. This analysis also shows that most of the oxygen functions are hydroxyl and epoxy groups but also carbonyl and carboxyl functions are present as minor constituents [35].

The structural characterization of G-(H_2_L) is important because it is assumed that there is a direct relationship between the catalytic behavior of G-(H_2_L)-Pd and the structural features of its components. 

**Figure 5:**
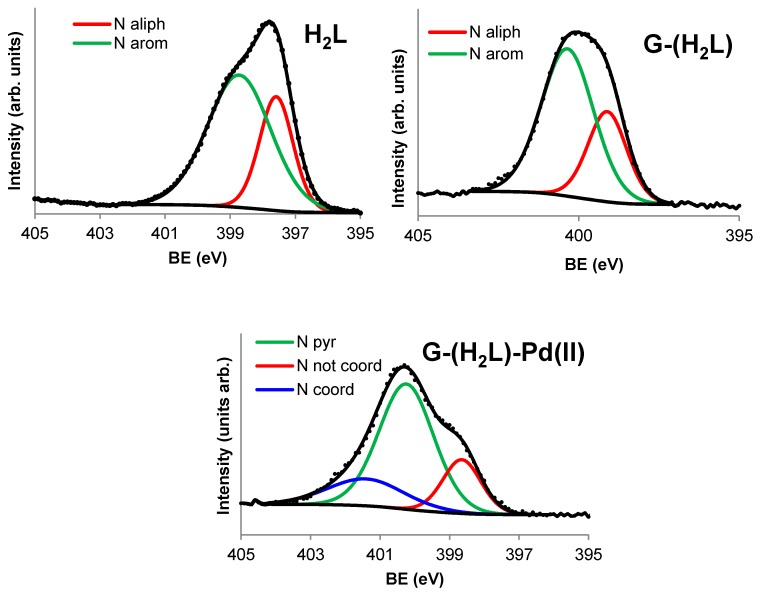
N1s signals of the X-ray photoelectron spectra (XPS) of H_2_L, G-(H_2_L) and G-(H_2_L)-Pd.

**Figure 6:**
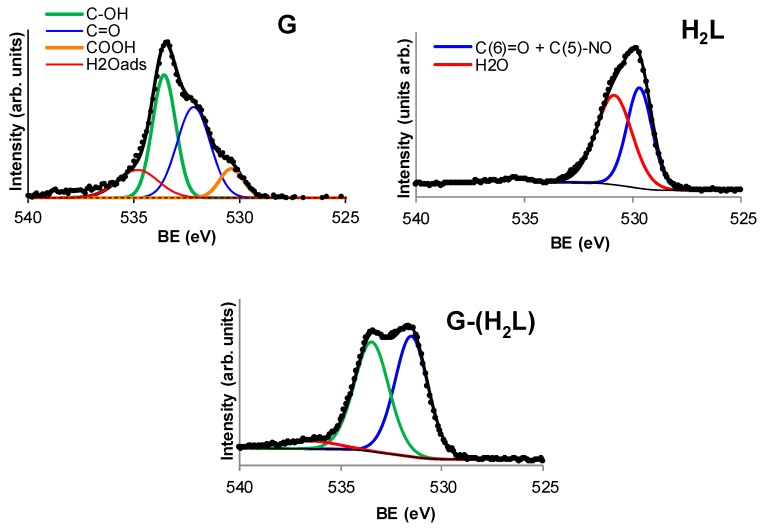
O1s signals of the XPS spectra of G, H_2_L and G-(H_2_L).

N1s and O1s components of the XPS spectrum of H_2_L and G-(H_2_L) are shown in Figure 5 and Figure 6, respectively. Comparison of these components provide valuable information on the way by which H_2_L interacts with G surface. XPS spectrum of H_2_L in the N1s range (Figure 5) exhibits a signal with two components at 397.6 eV and 398.7 eV, whose relative intensities, correspond to the number of aliphatic and aromatic nitrogen atoms of H_2_L (11 and 5, respectively). The shift to a BE value of 399.1 eV of the component of the aliphatic N atoms upon adsorption of H_2_L on G can be explained by considering that G-(H_2_L) was prepared in water at pH 5, where some protonation of the aliphatic amino groups occurs. The component due to the aromatic N atoms is also shifted to a significant higher binding energy (400.4 eV) in the XPS spectrum of G-(H_2_L), indicating a strong interaction of the pyrimidine moieties of H_2_L (containing ten N atoms) with the Cπ arene centers of G. Interaction with graphene-like surfaces of the pyrimidine moieties of similar ligands, that gave rise to shifting toward higher BE values of ring and conjugate nitrogen atoms, has been previously described as the result of π-π stacking of the pyrimidine plane with the Cπ ones (pyrimidine-Cπ interactions) [15,20]. This occurs because the interacting sites are compressed one on the other giving rise to local repulsion of the adjoined π clouds, which causes deshielding the N pyrimidine atoms. 

It is interesting to note that the energy of the N atoms of the two pyrimidine residues of the adsorbed H_2_L molecule are equivalent, which indicates that they interact in a similar way with the surface of G.

The O1s spectrum of pristine G (Figure 6), contains the components corresponding to COOH, C=O, C-OH and H_2_O functions [35] whose binding energies and intensities increase, respectively, between 530 eV and 535 eV. The O1s spectrum of H_2_L (Figure 6) contains a component at 529.7 eV which is assigned to C(6)=O and C(5)=NO groups of the two pyrimidine moieties of H_2_L and a second component a 530.8 eV corresponding to structural water of H_2_L (5 H_2_O per mole of H_2_L). In the O1s spectrum of G-(H_2_L), the 529.7 eV component of H_2_L is shifted to 531.0 eV, the pyrimidine-conjugate O atoms behaves similarly to the pyrimidine conjugate N atoms due to the above mentioned π-π interaction. 

**Figure 7:**
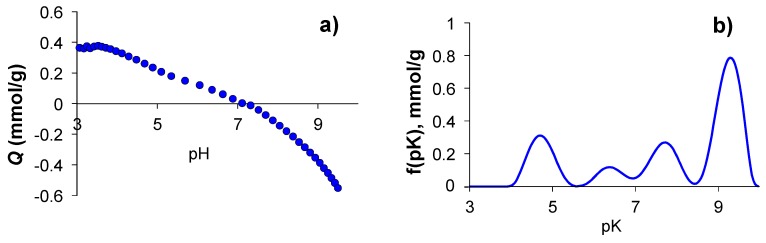
**Figure 7**. (**a**) Plot of surface charge density values of G-(H_2_L) (*Q* in mmol of H^+^ per gram) as a pH function and (**b**) profile of pKa values of the acidic of G-H_2_L as a pH function.

**Figure 8:**
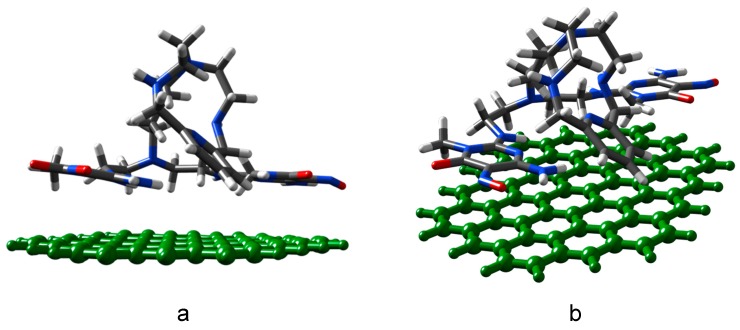
Lateral (**a**) and top (**b**) views of the minimum energy conformation of G-(H_2_L). Color code: grey, C; green, graphene C; white, H; red, O; blue, N.

The plots of the proton binding isotherm and the corresponding p*K*a versus pH of G have been reported previously [35] while those of G-(H_2_L) are shown in Figure 7. As said above, preparation of G-(H_2_L) was performed in water at pH 5.0 where protonation of the three secondary amine groups of H_2_L occurs (Figure 4). At the initial pH 3 of the titration experiments developed to obtain the proton binding isotherm, one of the two C(5)NO groups of the pyrimidine moieties should be partially protonated (c.a. 50%, according the acid-base behavior of H_2_L, see above). Accordingly, the positive surface charge density of G-H_2_L at such pH should be attributed both to the partial protonation of C(5)NO groups and to presence of H_3_O^+^ groups attached to the Cπ centers of G (Cπ-H_3_O^+^ adducts). The fact that the positive charge density of G-(H_2_L) (0.40 mmol of protons per gram) is lower than that on the G surface (0.45) at pH = 3.0 [35] points out that the interaction of the pyrimidine residues with the surface of G probably prevents the protonation of the co-planar C(5)NO groups, as previously observed with similar ligands [10]. Actually, in the SAIEUS plot no deprotonation steps are observed around a pKa value of about 3 assignable to the deprotonation of the C(5)-NO groups (see acid-base character of H_2_L). The surface charge decreases steadily in the 3.0-9.5 pH range (Figure 7) due to successive deprotonation steps involving both the acidic groups of G (anhydride groups at pH 4.6, Cπ-H_3_O^+^ at c.a. pH 7.8 and hydroxyl groups at c.a pH 9.3 [35]) and the protonated amino groups of H_2_L, namely: *i*) the aliphatic primary amino and the two (cyclic) secondary amino groups, *ii*) the C(2)_pyr_-NH groups conjugated with the pyrimidine residues. The p*K*a values for the corresponding protonation steps can be derived from Figure 7b. The asymmetric peak at c.a. pH 4.7, with relatively high intensity, contains two components corresponding to the anhydride groups of G and to the ligand amino group that has a protonation constant of log*K* = 5.35 in the free ligand (Table 1). Analogously, also the p*K*a values of 6.3 and 7.8 (Figure 7) corresponding to deprotonation of ligand amino groups of G-(H_2_L) are significantly lower than the corresponding values for the same amino groups of the free ligand. Such loss of basicity of ligand amine groups in G-(H_2_L), that was previously observed for similar materials [20], was rationalized in terms of different conformations of the ligand, in respect to the solution. An elongated arrangement is required to optimize the stacking interactions with G surface, hampering to some extent the protonation of such groups. This assumption was supported by theoretical calculations of the minimum energy for the interaction of H_2_L with an idealized G surface (see experimental section). The results showed that H_2_L assumes a conformation in which both pyrimidine rings give rise to a plane-to-plane stacking interaction with the surface of G, while the aliphatic ligand chain actually adopts an elongated conformation resulting from the contact of >CH pyridine groups with the G surface (Figure 8).

Finally, the intense peak at c.a. pH 9.3 which moderately overlaps with the peak at pH 7.8 (Figure 7b) would contains the components due to deprotonation of the Cπ-H_3_O^+^, the hydroxyl surface functions of G and probably part of the C(2)-NH_pyr_ groups of H_2_L (Figure 4).

### 2.4. Preparation and Characterization of the G-(H_2_L)-Pd Hybrid

The G-(H_2_L)-Pd catalyst was prepared by mild adsorption of PdCl_4_^2-^ in water solution (pH 5) on solid G-(H_2_L) containing 0.48 mmol of H_2_L per gram of G. The resulting G-(H_2_L)-Pd hybrid contained 0.60 mmol of Pd per gram of the final product. This value, compared with the 0.15 mmol adsorbed by 1g of the bare G under the same experimental conditions, is indicative of the coordination effect of the H_2_L functionality in retaining Pd^2+^ by G-(H_2_L). Evidently, the complexing ability of H_2_L is higher than the ability of the bare G to adsorb PdCl_4_^2-^ on its surface through a primary interaction of this planar anion with the Cπ centers of G [17]. A similar behavior was previously observed in the case of hybrids obtained by adsorption of ligands similar to H_2_L on a MWCNT [20].

A close inspection of the XPS spectrum of G-(H_2_L)-Pd(II) shows that the N1s signal contains three components (Figure 5). One of them is peaked at 400.5 eV and corresponds to the N atoms of the pyrimidine rings and to those of the two pyrimidine-conjugated C(2)-NH_pyr_ groups. This BE is similar to the corresponding value for G-(H_2_L), indicating that the coordination of Pd^2+^ does not alter the stacking interaction of pyrimidine moieties of the anchored ligands with the surface of G. A shoulder with intensity corresponding to two N atoms, at a BE value of 399.2 eV, which is similar to that found for the aliphatic N atoms in the case of the G-(H_2_L) hybrid, would correspond to three amino groups that do not take part in the coordination to Pd^2+^. A shoulder of the main peak at the highest BE value (c.a. 401.5 eV), with intensity corresponding to three N atoms of H_2_L, can be assigned to the N atoms coordinated to Pd^2+^, namely, two secondary amino and the pyridine N atoms. The observed shifting of the signal of these atoms to higher BE values, relative to their positions in the G-(H_2_L) spectrum (399.1 eV, see above), stems from the electron donation to Pd^2+^ ions.

Under the assumption that polyamine functions of G-(H_2_L) are prevalent adsorption sites for Pd^2+^ compared to the Cπ centers of G, of the 0.6 mmol of Pd(II) contained in 1g of G-(H_2_L)-Pd, 0.48 mmol would be complexed by the polyamine and the remaining, c.a. 0.12 mmol, would be adsorbed on Cπ centers of G. 

**Figure 9:**
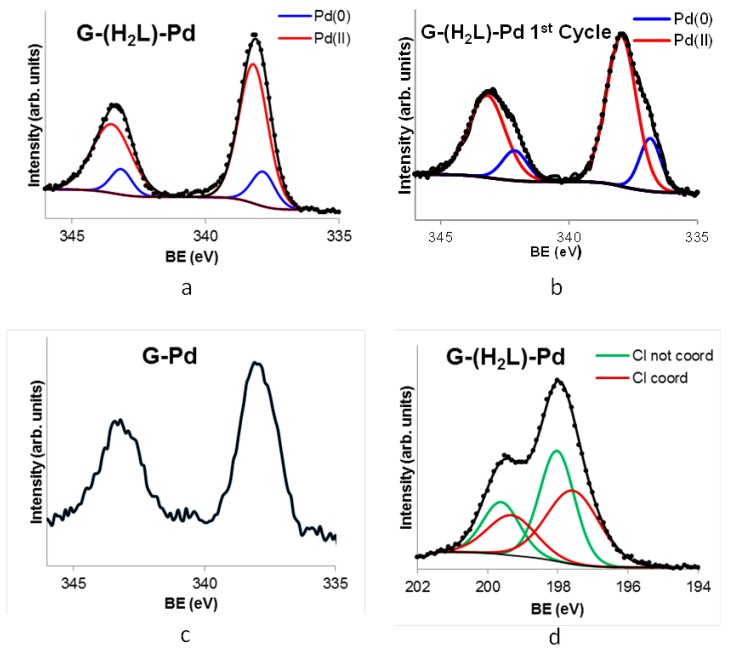
High-resolution XPS spectra of G-(H_2_L)-Pd: fresh (**a**) and after the first reaction cycle (**b**), of G-Pd in the Pd 3d_5/2_ and Pd 3d_3/2_ regions (**c**) and spectra of G-(H_2_L)-Pd in the Cl 2p_3/2_ and Cl 2p_1/2_ regions (**d**).

**Figure 10:**
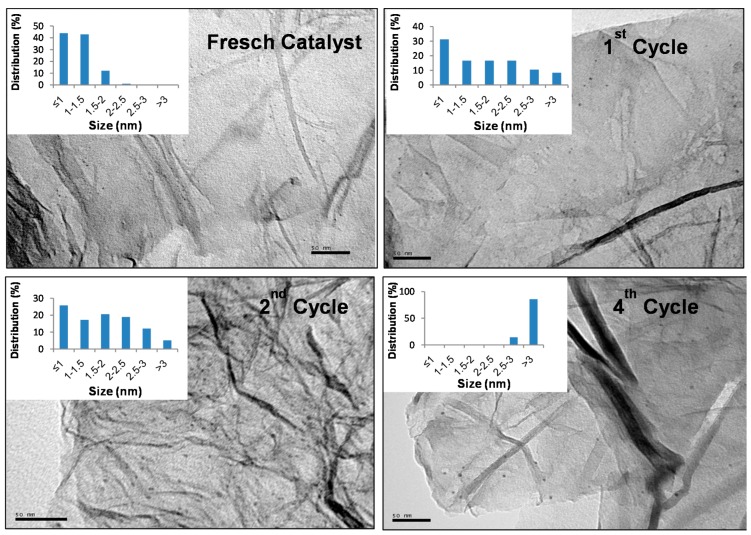
Selected transmission electron microscopy (TEM) images of the fresh and the reused catalysts after 1st, 2nd and 4th cycles and plots of the distribution sizes of Pd^0^ nanoparticles (insets).

The Pd 3d5/2 and Pd 3d3/2 XPS spectrum of G-(H_2_L)-Pd (Figure 9) exhibits two slightly asymmetric signals with maxima at 343.5 eV and 338.5 eV which are characteristics of Pd^2+^. On the other hand, the XPS spectrum of G-Pd (see above) shows the presence of a residual amount of chlorine (the atomic percent relationship %Cl/%Pd < 1), which means that most of palladium adsorbed on the bare G is reduced to Pd^0^. This suggests that in G-(H_2_L)-Pd, although the majority of palladium is present as Pd^2+^ (c.a. 80%), a residual part (c.a. 20 %) adsorbed on the surface of G is in the form of Pd^0^. Indeed, TEM images obtained from a G-(H_2_L)-Pd sample (Figure 10, fresh catalyst) show the presence of very small-sized Pd^0^ nanoparticles uniformly spread on the G surface. 

Calculations based on the Pd and Cl signals in the XPS spectra of G-(H_2_L)-Pd afford a Cl/Pd molar ratio, relative to Pd^2+^ coordinated to the polyamine (c.a. 80% of all Pd), very close to 2. Furthermore, each of the two peaks assigned to electrons from 2p_3/2_ and 2p_1/2_ states of Cl includes two components (Figure 9d) pertaining to the differently bound chloride ions. This finding is in agreement with the above observations pointing out that H_2_L acts as a tridentate ligand toward Pd^2+^ to form a complex in which the metal ion completes its square-planar geometry through the coordination of a Cl^-^ anion and necessitates a second Cl^-^ to ensure charge neutrality. Accordingly, the components at lower and higher B.E. values in the XPS spectrum can be ascribed, respectively, to non-coordinated and coordinated chloride ions.

Summarizing the information in this section, we can say that H_2_L is adsorbed on G giving rise to a hybrid material in which the ligand is anchored via π-π stacking interaction of both its pyrimidine moieties with Cπ centers of G. It is also observed a decreasing of the basicity of both aliphatic and pyridine amino groups. This is probably due to an elongated arrangement of the polyamine, adopted to optimize the stacking interactions, which somehow hampers the basicity of the amino groups. Nevertheless, the adsorption of Pd^2+^ on the G-(H_2_L) takes place mainly via metal coordination to these polyamine functions, leading to a G-(H_2_L)-Pd hybrid consisting of a uniform distribution on the G surface of H_2_L-Pd^2+^ complexes anchored by the two pyrimidine groups.

### 2.5. Catalytic Activity of G-(H_2_L)-Pd in the Sonogashira Carbon-Carbon Coupling Reaction

For comparison purposes, the catalytic activity of G-(H_2_L)-Pd in the copper-free Sonogashira reaction between iodobenzene (IB) and phenylacetylene (PA) was tested under the same condition previously used [20] for MWCNT-(HL1)-Pd (IB/PA molar ratio equal to 1/1.5, water as the solvent, triethylamine as the base, aerobic conditions, 50 °C). Also, in the present case, the working temperature was chosen taking into account the low solubility in water of the reactive substrates at lower temperatures and the possible lixiviation of the anchored (H_2_L)-Pd complex at higher ones. 

At this temperature, MWCNT-(HL1)-Pd behaved as an efficient catalyst in the above reaction, yielding 90% of conversion of the reactants in the first reaction cycle after a very short equilibrium time of 2 h. This result was attributed to the easy accessibility of the catalytic active centers, the anchored Pd-HL1 complexes, which were homogeneously dispersed over the whole external surface of the MWCNTs [20]. Considering the similarity of the G and MWCNT surfaces, we expected a similar behavior with our new G-(H_2_L)-Pd catalyst and, possibly, an even better performance, at least in term of catalyst stability, due to the double-anchorage of the complex on the surface of G. 

Indeed, results showed that the intensities ratios of C1s and N1s XPS signals of the fresh G-(H_2_L)-Pd catalyst is maintained after use, indicating that no lixiviation of H_2_L occurs during the reaction, in contrasts to the non-negligible release of HL1 observed [20] under the same conditions from MWCNT-(HL1)-Pd. That is, the double anchorage to the graphitic surface has a favorable stabilizing effect. Furthermore, adopting reaction conditions similar to those previously used for MWCNT-(HL1)-Pd, a similar catalytic activity was obtained for G-(H_2_L)-Pd in terms of reaction products (diphenylacetylene) and reaction yields (c.a. 90%) but the equilibrium time (14 h) was significantly longer. 

**Figure 11:**
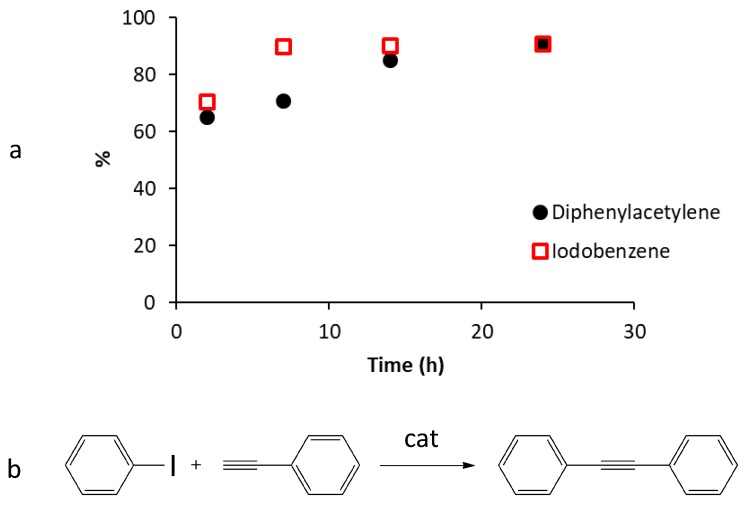
(**a**) Amounts of the formed diphenylacetylene, DAC, (referred to the theoretical 100 % in mmol) and of the consumed iodobenzene, IB, (referred to initial 100 % in mol) as a time function in the course of the reaction of iodobenzene with phenylacetylene. (**b**) The studied Sonogashira cross-coupling reaction.

The reaction course was followed by monitoring the amounts of IB present in solution as a function of time, which revealed that the concentration of this reactant at certain reaction times was significantly lower than that corresponding to the amounts of diphenylacetylene formed. This fact, which is illustrated in Figure 11, suggests that IB is partly adsorbed on the surface of G and its desorption is likely a limiting step for the reaction kinetic. Probably, the same happens for PA. Apparently, the planar surface of G favors a stronger interaction with the aromatic reactants in the case of G-(H_2_L)-Pd with respect to the curved surface of MWCNT in MWCNT-(HL1)-Pd. 

It is worth mentioning that blank experiments showed that neither G nor G-(H_2_L) exhibit any catalytic activity, whereas a G-Pd sample containing 1 % by weight of Pd gave, under the above experimental conditions, a yield of c.a. 60 % after 14 h.

In addition to adsorption phenomena, also structural changes that the fresh G-(H_2_L)-Pd catalyst could have suffered during the process could have affected its catalytic efficiency. As shown by the XPS spectra of G-(H_2_L)-Pd samples obtained after use in the catalytic reaction, a significant metal lixiviation occurs, the Pd content diminishing from 0.60 mmol per gram of G-(H_2_L) (in the fresh catalyst) to c.a. 0.3 mmol per gram (Figure S3). This loss far exceeds the amount of Pd^0^ deposited on the surface of G in the fresh G-(H_2_L)-Pd catalyst (0.12 mmol per gram, see above) pointing out that also part of Pd^2+^ coordinated to H_2_L is lixiviated. Moreover, TEM images of the surface of the used G-(H_2_L)-Pd material show that a significant portion of the palladium remaining on the surface of G is in the form of Pd^0^ nanoparticles with mean sizes significantly greater than those in the fresh catalyst (Figure 10). In agreement with this observation, the signals corresponding to Pd 3d5/2 and Pd 3d3/2 in the XPS spectrum of the used G-(H_2_L)-Pd recovered after the first catalytic cycle (Figure 9b) are evidently broadened, relative to the fresh catalyst, in agreement with an increased reduction of Pd^2+^. 

According to the proposed mechanism for the copper-free Sonogashira reaction [36], the partial reduction, occurring during the reaction, of Pd^2+^ coordinated to H_2_L is not surprising and should be related to the formation of a Pd^0^ complex as an intermediate species of the catalyzed process. This effect, which was insignificant in the case of MWCNT-(HL1)-Pd [20] is probably favored for the G-(H_2_L)-Pd catalyst due, as above said, to an elongated arrangement of the polyamine, facing the surface of G, which is adopted to optimize the stacking interactions, thus hampering the complexing ability of the polyamine to Pd^2+^, that is, the stability of the coordination of the polyamine to Pd(II). Two additional important results deserve to be mentioned. (i) The ratio between the intensities of C1s and N1s signals of the fresh G-(H_2_L)-Pd catalyst is equal to that of the used catalyst, in contrast with the results previously obtained with MWCNT-(HL1)-Pd for which a significant release of HL1 was observed. This means that, regarding lixiviation phenomena, G-(H_2_L)-Pd is more stable than MWCNT-(HL1)-Pd, as expected because of the double-anchorage of H_2_L to the substrate surface. (ii) The XPS spectrum in the N1s range of the fresh G-(H_2_L)-Pd catalysts does not change after use (Figure S4), strongly suggesting that the amine groups of H_2_L are probably involved in retaining the Pd^0^ nanoparticles deposited on the surface of G. This is an important fact related to the structural stability of the catalyst used; in fact, after the first use, the catalyst G-(H_2_L)-Pd does not undergo further structural modifications in the subsequent reaction cycles (see below).

As shown by XPS analysis, after the first use, the composition of the catalyst during three additional cycles of the catalytic reaction remains almost constant. Only a little increase in the weight percent of C was found, which is attributed to a modest accumulation of reactants and diphenylacetylene on the catalyst surface, while the amounts of nitrogen and Pd remain practically unchanged (Figure S3). Moreover, the deconvolution of Pd 3d5/2 and Pd 3d3/2 signals of the reused catalyst also reveal that there are no substantial changes of the Pd^2+^/Pd^0^ ratio after each reaction cycle (Figure S5). These are further evidences of the high stability of the G-(H_2_L)-Pd hybrid material recovered after the first reaction cycle, namely of the efficiency of H_2_L anchoring on the surface of G and of the role played by H_2_L in the stabilization of both Pd^2+^ and Pd^0^ catalytic centers.

Despite this, the efficiency of the reused catalyst decreases steadily over the three successive reaction cycles. Kinetic reaction data indicate that the maximum efficiency is reached after 7 h: the formation of diphenylacetylene (DCA) constantly increases from the start of the reactions up to 7 h later to successively decrease during the following 18 h. Most likely, this is due to the adsorption of DCA on the catalyst which over time prevails over its formation. The yield of the reaction after 7 h, in four reaction cycles, varies from 72 % in the first cycle, to 69 %, 63 % and 50 %, respectively, in the following three. This clear deactivation of the catalyst during its reuse can be attributed to the constant agglomeration of Pd^0^ nanoparticles, occurring during subsequent reuse cycles (Figure 10), which reduces the number of active Pd^0^ catalytic centers.

## 3. Materials and Methods

### 3.1. General

All starting materials were high purity compounds purchased from commercial sources and were used as supplied. Compounds **1** (6-[7-(diaminoethyl)-3,7-diazaheptyl]-3,6,9-triaza-1-(2,6-pyridina)-cyclodecaphane) [37] and 6-amino-3,4-dihydro-3-methyl-2-methoxy-5-nitroso-4-oxopyrimidine (**2**) [38] were prepared according to described procedures.

### 3.2. Synthesis of the Ligand

The ligand H_2_L was prepared from the precursor **1** (Figure 2) by reaction with **2**. 0.66 g (1.55 mmol) of **1** was dissolved in 75 cm^3^ of methanol and reacted at room temperature with 0.62 g (3.35 mmol) of **2**. The latter dissolves slowly over 6 h under stirring at room temperature. The mixture was then heated to 45 °C under stirring for 3 h. After that, 1 cm^3^ of 37% NH_3_ was added to convert the excess of **2** into the insoluble 2,4-diamino-1-methyl-5-nitroso-6-oxopyrimidine derivative at room temperature. The solution was then evaporated to half of the original volume and left overnight at 10˚C. The resulting suspension was filtered, and the solution was evaporated to dryness under vacuum at room temperature to obtain H_2_L as a deep purple solid compound. Yield 82%. ^1^H-NMR (300 MHz, D_2_O) δ 7.91 (1H, t, *J* = 8 Hz), 7.41 (2H, d, 8 Hz), 4.59 (4H, s), 3.85 (2H, m), 3.60 (4H, m), 3.22 (16H, m), 3.02 (4H, m), 2.88 (4H, m), 2.20 (2H, m). ^13^C-NMR (75 MHz, D_2_O) δ 161.34, 155.78, 152.06, 148.83, 139.72, 137.84, 122.12, 81.70, 51.92, 50.91, 50.42, 49.47, 45.84, 44.83, 43.74, 36.76, 28.04. MS (ESI^+^): *m*/*z*: 697.41 [M + H]^+^. Anal. calcd. for C_30_H_48_N_16_O_4_∙5H_2_O: C, 45.8; H, 7.4; N, 28.5. Found: C, 46.0; H, 6.9; N 28.3.

### 3.3. Synthesis of [Pd_2_(H_2_L)Cl_4_]∙(CH_3_OH)_2_∙2.5H_2_O

A solution of K_2_PdCl_4_ (20.1 mg, 0.062 mmol) in water (1 cm^3^) was added to a boiling solution of H_2_L (48.9 mg, 0.062 mmol) in methanol (5 cm^3^). The resulting solution was left to evaporate to dryness at room temperature, then the resulting solid was dissolved in methanol (3 cm^3^) and added with butanol (1 cm^3^). The solid complex was formed upon slow evaporation of this solution at room temperature. The complex was collected by filtration, washed with diethyl ether and dried in the air at room temperature. Yield 65%. Anal. calcd. for C_30_H_48_N_16_O_4_PdCl_2_ ([Pd(H_2_L)]Cl_2_): C, 41.22; H, 5.53; N, 25.64. Found: C, 41.01; H, 5.47; N, 25.53.

### 3.4. Potentiometric Measurements

Potentiometric (pH-metric) titrations, employed to determine the protonation constants of the studied ligand and of its Pd(II) complex, were performed in 0.15 M NaCl at 298.1 ± 0.1 K using an automated apparatus [39] and a procedure [40] previously described. The combined Metrohm 6.0262.100 electrode was calibrated as a hydrogen-ion concentration probe by titration of previously standardized amounts of HCl with CO_2_-free NaOH solutions and determining the equivalent point by Gran’s method [41], which gives the standard potential, E° and the ionic product of water (p*K*_w_ = 13.73(1) in 0.15 M NaCl at 298.1 K). The computer program HYPERQUAD [42] was used to calculate the stability constants from the potentiometric data. The concentration of ligands was about 1 × 10^−3^ M in all experiments. The studied pH range was 2.5–11.0. At least two measurements were performed for each system: at first, the different titration curves were treated as separated curves without significant variations in the values of the calculated stability constants; finally, the sets of data were merged together and treated simultaneously to give the final stability constants. Although it was not possible to determine the equilibrium constants for the formation of [Pd(H_2_L)]^2+^ complex due to the slowness of the complexation reaction, these species proved to be very stable once formed and could be titrated in the pH range 2.5–8.5 according to fast protonation equilibria. At pH higher than 8.5, precipitation of the complex occurred. Titrations were performed by means of the above method with solutions containing H_2_L and K_2_PdCl_4_ (1 × 10^−3^ M) equilibrated for 5 days at pH 3 and 298 K, thus making possible the determination of the protonation constants of the complexes. Different equilibrium models for the studied systems were generated by eliminating and introducing different species. Only those models for which the HYPERQUAD program furnished a variance of the residuals σ^2^ < 9 were considered acceptable. Such a condition was unambiguously met by a single model for each system. 

### 3.5. Preparation of the Catalyst G-(H_2_L)-Pd

The graphene, G, used as carbon-support in the preparation of the G-(H_2_L)-Pd hybrid, was purchased from NanoAmor (Katy, TX, USA). According to the manufacturer, the product has a carbon content >98wt%, a size between 2 and 10 µm and 1–3 layers. Before using, the commercial solid was repeatedly washed with double distilled water and dried in the air until constant weight. The composition of G after washing was C (94.8%) and O (4.6%), as the main constituents according to X-ray photoelectron spectroscopy (XPS) analysis (Kratos Analytical, Manchester, UK). 

The G-(H_2_L)-Pd material was prepared by a two-step procedure. Firstly, H_2_L was adsorbed on G. Preliminary adsorption experiments were conducted in water, at three days equilibrium times with various initial concentrations of H_2_L and pH. From these experiments it emerged that the maximum adsorption capacity of H_2_L on G (0.5 mmol of H_2_L per gram of G) takes place at pH 5. To obtain the G-(H_2_L) material used to prepare the catalysts, 0.097 g of G were mixed with 390 cm^3^ of 7.5 × 10^−4^ M aqueous solution of H_2_L at pH 5 in a plastic flask. The flask was shaken in an air-thermostated bath at 298.1 K until the adsorption equilibrium was reached (3 days). The obtained G-(H_2_L) solid was separated by filtration, repeatedly washed with distilled water and dried into a desiccator. The final amount of adsorbate per gram of adsorbent was quantified in 0.48 mmol of H_2_L per gram of G.

In the second step, the G-(H_2_L)-Pd catalyst was prepared by adsorption of Pd^2+^, from K_2_PdCl_4_, on the appropriate G-(H_2_L) material. Accordingly, 0.1 g of G-(H_2_L) was mixed in a suitable plastic flask with 100 cm^3^ of a 1 M KCl aqueous solution containing K_2_PdCl_4_ 5 × 10^−4^ M and the pH was adjusted to 5.0 through the addition of aqueous HCl. pH 5.0 was selected as the best compromise between minimizing the proton competition with Pd^2+^ coordination to the ligand amino groups and preventing the formation of Pd^2+^ hydroxy-species that, at this pH value, are not yet formed (i.e., all the Pd^2+^ is present as [PdCl_4_]^2−^ [10]). The suspension was shaken in an air-thermostated bath at 298.1 K during four days until the adsorption equilibrium was reached (i.e., until the UV absorbance of the Pd^2+^ solution at λ = 474 nm remained constant over time). Finally, the amount of adsorbed Pd was determined as the difference between the initial and the final absorbances at λ = 474 nm: 0.60 mmol Pd per gram of G-(H_2_L).

### 3.6. Determination of the Surface Charge Density of G-(H_2_L)

The surface charge density (*Q* in mmol of H^+^ per g of adsorbent) of the G-(H_2_L) material was determined by a method based on potentiometric titration data [43,44] according to an already described procedure [18].

*Q* was calculated by means of Equation (1):

(1) $$Q=\frac{1}{m}\left(V_{0}+V_{t}\right)\left({\left[H\right]}_{i}-{\left[OH\right]}_{i}-{\left[H\right]}_{e}+{\left[OH\right]}_{e}\right)$$

where *V*_0_ and *V_t_* are the volumes of initial solution and titrant, respectively and *m* is the mass of the adsorbent. Subscripts *i* and *e* refer to the initial and equilibrium concentration of protons or hydroxyl ions. The proton isotherms are obtained by plotting *Q* versus equilibrium pHs.

### 3.7. Spectroscopic Measurements

Absorption spectra were recorded at 298 K on a Jasco V-670 spectrophotometer (JASCO Analitica, Madrid, Spain). Both ligand and metal complex solutions were about 5 × 10^−5^ M. Complex solution (1:1 metal:ligand molar ratio) were prepared by adding the appropriate amount of a standardized PdCl_4_^2−^ solution to a ligand solution at pH 3. The solution was left to equilibrate at 298 K for 5 days. 5 cm^3^ samples of this solution were taken in separated vials and their pH was adjusted at different values within the 0.9–11.5 pH range by addition of HCl or NaOH solutions. These samples were stored at 298 K and their absorption spectra were recorded daily: no variations were observed after two days. Also, the pH of the equilibrated samples was recorded. The ^1^H-NMR spectra (300 MHz) in D_2_O solution were recorded at 298 K on a Bruker Advance 300 (Bruker Spain, Madrid, Spain). The XPS spectra of solids were recorded with a Kratos Axis Ultra DLD spectrometer. Monochromatic Al/MgKα radiation from twin anode in constant analyzer energy mode with pass energy of 160 and 20 eV (for the survey and high resolution spectra, respectively) was used. The C1s transition at 284.6 eV was used as a reference to obtain the heteroatoms binding energies. The accuracy of the binding energy (BE) values was ±0.2 eV.

### 3.8. Elemental Analyses of G-Based Solids

The elemental analyses of the G-based solids of this work were obtained from the quantitative analysis of the corresponding high-resolution XPS spectra. The use of XPS spectra for the quantitative elemental analysis of solids is limited when the sizes of the particles are bigger than a few nanometers, since the penetrating power of the radiation used in this technique is also limited. However, the small thickness of the particles of the solids studied in this work (which are formed by stacking of a maximum of three G sheets, that is, less than 1 nm thick, see above) allows reliable quantification of the elemental components using data from the corresponding high resolution XPS spectra. The counting of Pd^0^ nanoparticles in the solids was carried from digital electron micrographs (TEM images. Jeol Equipement, Akishima, Tokyo, Japan), following the protocol established in The Particle Size Analysis program [45]. 

### 3.9. General Procedure for the Sonogashira Reaction

A mixture of iodobenzene (1 mmol), phenylacetylene (1.5 mmol), Et_3_N (2 mmol), H_2_O (1 cm^3^) and the catalyst (10 mg of G-(H_2_L)-Pd) was stirred under aerobic conditions at a constant temperature (50 or 70 °C). The progress of the reaction was monitored by gas chromatography (GC). After completion, CHCl_3_ (10 cm^3^) was added to the reaction mixture and the catalyst was recovered by filtration and washed with CHCl_3_ (2 × 5 cm^3^) and H_2_O (2 × 5 cm^3^). The organic layers were collected and dried over anhydrous Na_2_SO_4_. The analysis of the reaction products in the organic phase was performed by GC using a 7820A Agilent GC System chromatograph, with an Agilent 190915-433 column, 30 m × 250 µm × 25 µm and a flame ionization detector (FID) (Agilent, Santa Clara, CA, USA). The recovered catalyst was reused for another batch of the same reaction. The process was repeated for three additional runs.

### 3.10. TEM Micrographs 

TEM images of G-(H_2_L)-Pd samples were obtained with a JEOL Mod. JEM-1010 equipment (Jeol Equipement, Akishima, Tokyo, Japan).

### 3.11. Theoretical Calculations of Optimum Geometry

Theoretical calculations of the optimum geometry of the G-(H_2_L) adduct were derived from the conformation corresponding to the global minimum in vacuum by using the AMBER algorithm [46] of the Gaussian09 program suite [47]. The most stable structure (with minimal interaction energy) was considered as the optimal (equilibrium) structure. The final RMS gradient was less than 0.001 kcal mol^-1^ Å^-1^ for the minimized structure. Initially, the H_2_L molecule was placed above the surface of graphene at physisorption distances with their pyridines rings oriented parallel and perpendicular to the graphene surface, respectively, forming a “flat” (face-to-face) and an “edge” (edge-to-face) configuration. The system energy was minimized with a steepest descent algorithm [48]. Finally, the global minimum in interaction energy landscape and the corresponding structure were considered as the interaction energies and the equilibrium structure of the G-(H_2_L) adduct.

## 4. Conclusions

The supramolecular approach for catalyst preparation adopted in this work is confirmed as a promising criterion for the design of green, sustainable and energy-saving chemical processes. The catalyst G-(H_2_L)-Pd is constructed via two successive self-assembly processes that spontaneously develop under mild conditions (water, room temperature, aerobic atmosphere). G-(H_2_L)-Pd exhibits higher robustness than the reference MWCNT-(HL1)-Pd catalyst thanks to the double-anchoring of H_2_L to the surface of G compared to the single-anchoring of HL1 to MWCNTs. Moreover, the flat surface of G favors a more efficient interaction with the planar pyrimidine groups with respect to the curved surface of MWCNTs. Both catalysts behave with similar high efficiency (both yield up to 90% conversion in the first reaction cycle) in the copper-free Sonogashira –C-C- coupling reaction between iodobenzene and diphenylacetylene. Reusing the catalysts in three additional cycles results in a constant loss of efficiency, even if due to different causes. In the case of MWCNT-(HL1)-Pd the loss of activity was determined as due to progressive lixiviation of (HL)-Pd1 during reuse, which results in the loss of active catalytic centers [20]. In the case of G-(H_2_L)-Pd, the stronger interaction of H_2_L with the surface of G preserves the (H_2_L)-Pd complexes from lixiviation and the loss of activity runs parallel to the progressive agglomeration of the Pd^0^ nanoparticles formed in the first reaction cycle. Indeed, the Pd^2+^→Pd^0^ reduction which was not observed in the case of MWCNT-(HL1)-Pd is probably due to the interaction of the pyridine group of the macrocyclic pendant with the planar G surface which probably weakens the stability of the (H_2_L)-Pd complex. It is worth noting that also the increased adsorbance of the reagents on the flat surface of G has a significant effect on the catalytic efficiency of G-(H_2_L)-Pd, both lengthening the equilibrium time and reducing the reaction yields. 

To complete the picture provided by the HL1 and H_2_L ligands and the MWCNT and G support substrates, the next step will be to study the catalytic properties of the hybrid materials corresponding to the ligand-substrate combinations that have not yet been considered, that is, the MWCNT-(H_2_L)-Pd and G-(HL1)-Pd catalysts.

## Figures and Tables

**Table 1:** Protonation constants of the H_2_L ligand and of its Pd^2+^ complex determined in 0.15 M NaCl aqueous solutions at 298.1 ± 0.1.

	Log *K*
HL^-^ + H^+^ = H_2_L	10.85(2)
H_2_L + H^+^ = H_3_L^+^	9.27(2)
H_3_L^+^ + H^+^ = H_4_L^2+^	8.22(2)
H_4_L^2+^ + H^+^ = H_5_L^3+^	7.46(3)
H_5_L^3+^ + H^+^ = H_6_L^4+^	5.35(1)
H_6_L^4+^ + H^+^ = H_7_L^5+^	3.13(1)
H_7_L^5+^ + H^+^ = H_8_L^6+^	1.75(1)
Pd(H_2_L)^2+^ + H^+^ = Pd(H_3_L)^3+^	7.30(8)
Pd(H_3_L)^3+^ + H^+^ = Pd(H_4_L)^4+^	5.1(1)
Pd(H_4_L)^4+^ + H^+^ = Pd(H_5_L)^5+^	2.7(1)
Pd(H_5_L)^5+^ + H^+^ = Pd(H_6_L)^6+^	2.4(1)

## Supplementary Materials

The following are available online at https://www.mdpi.com/1420-3049/24/15/2714/s1, Distribution diagram of the Pd^2+^ complexes; UV spectra of H_2_L at different pH values; XPS surveys of fresh G-(H_2_L)-Pd and after 1-4 catalytic cycles; XPS spectra in the N1s region of G-(H_2_L)-Pd after 1-4 catalytic cycle; high-resolution XPS spectrum in the Pd 3d_5/2_ and Pd 3d_3/2_ regions of G-(H_2_L)-Pd after the fourth reaction cycle.
