# Supplementary material for: A New Heterogeneous Catalyst Obtained via Supramolecular Decoration of Graphene with a Pd2+ Azamacrocyclic Complex
Source: Molecules. 2019 Jul 26;24(15):2714. doi: 10.3390/molecules24152714 (PMC6696290; doi:10.3390/molecules24152714)
Supplement: Supplementary file 1 [file molecules-24-02714-s001.pdf]

Supplementary Materials

A new heterogeneous catalyst obtained via supramolecular decoration of graphene with a Pd^{2+} azamacrocyclic complex.

By: Matteo Savastano, Paloma Arranz-Mascarós, Maria Paz Clares, Rafael Cuesta, Maria Luz Godino-Salido, Lluis Guijarro, Maria Dolores Gutiérrez-Valero, Mario Inclán, Antonio Bianchi, Enrique García-España, and Rafael López-Garzón,

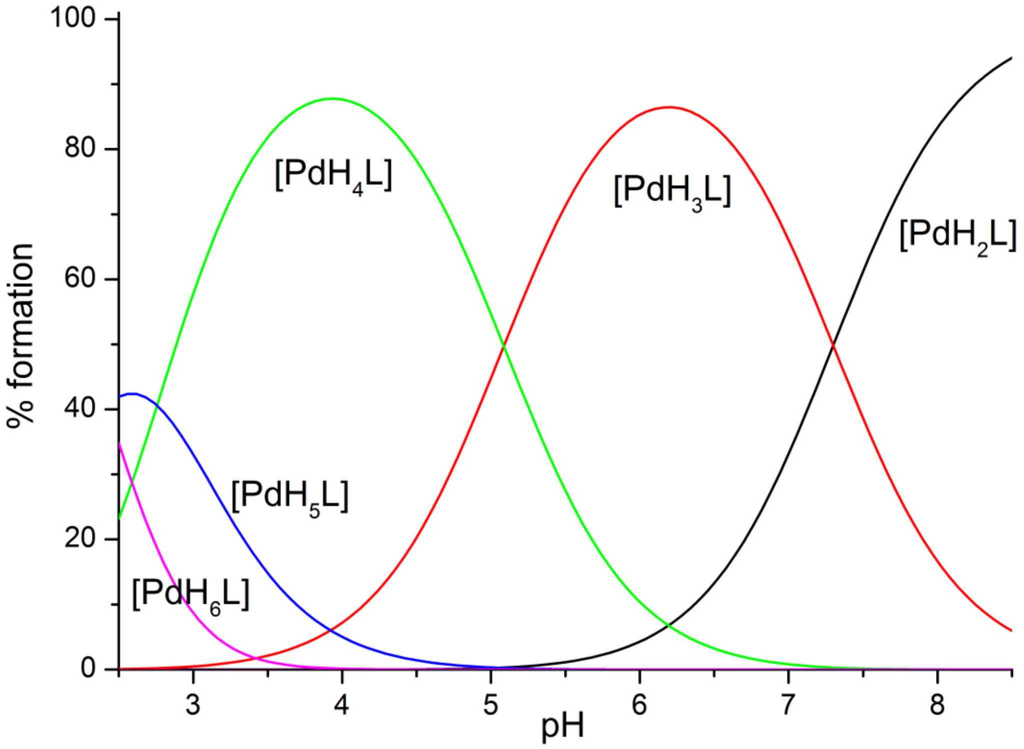

Figure S1. Distribution diagram of the Pd^{2+} complexes formed by H_2L as a function of pH. $[Pd^{2+}]$ = $[H_2L]$ = 1×10^{-3} M. Charges omitted.

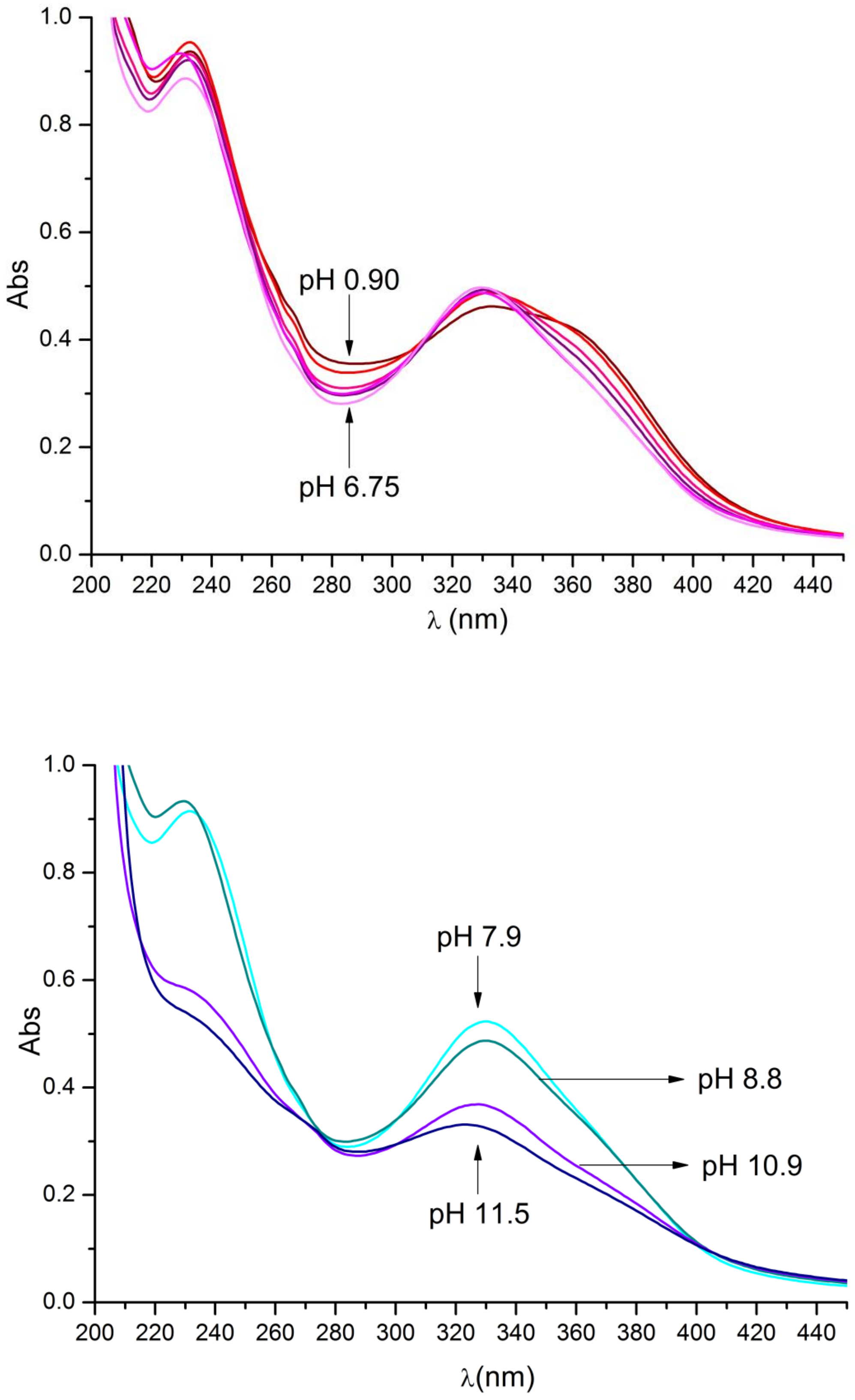

Figure S2. UV spectra of H_2L at different pH values.

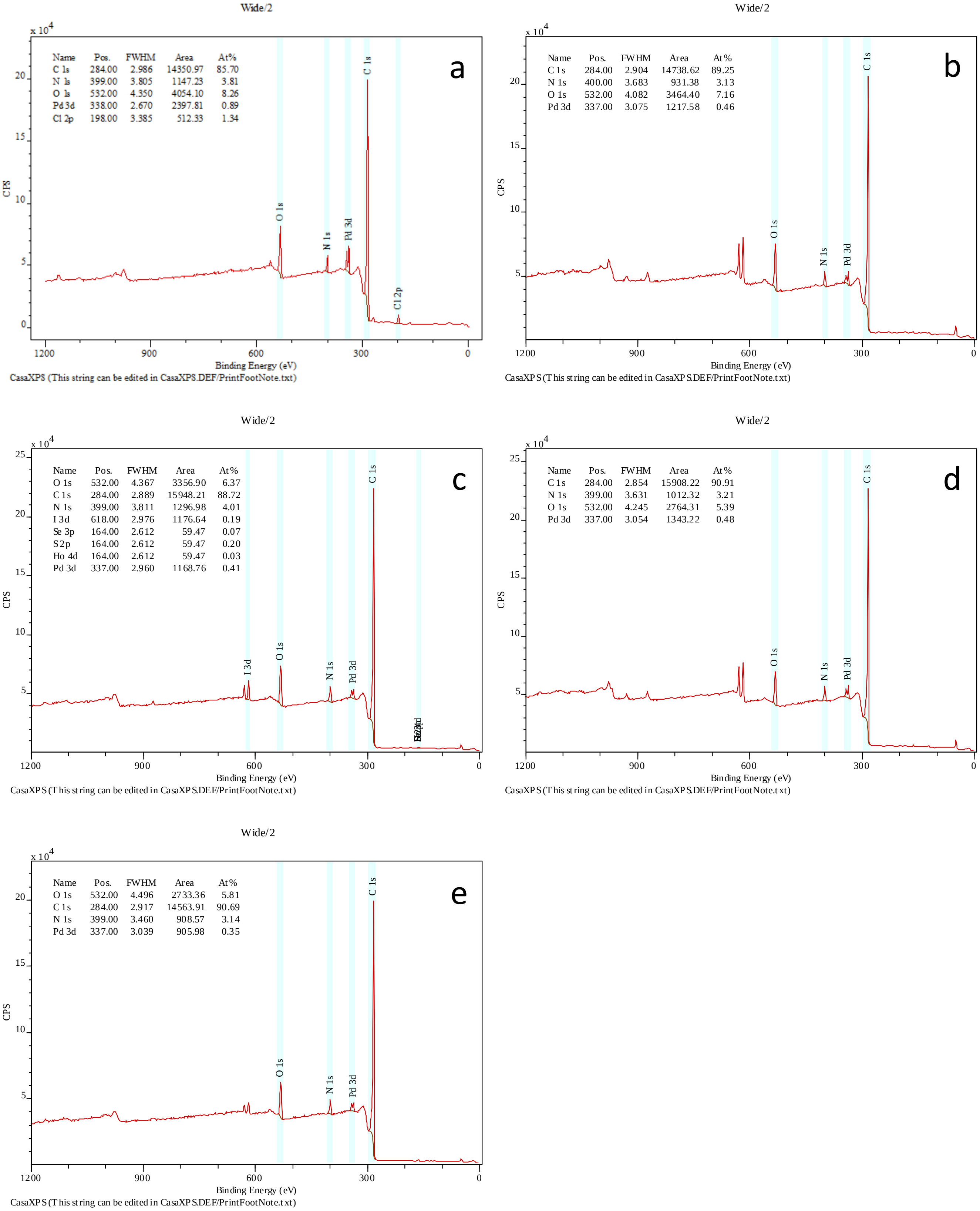

Figure S3. XPS survey (with areas) of fresh G-(H_2L)-Pd catalyst (a) and of the same after 1 (b), 2 (c), 3 (d) and 4 (e) catalytic cycles.

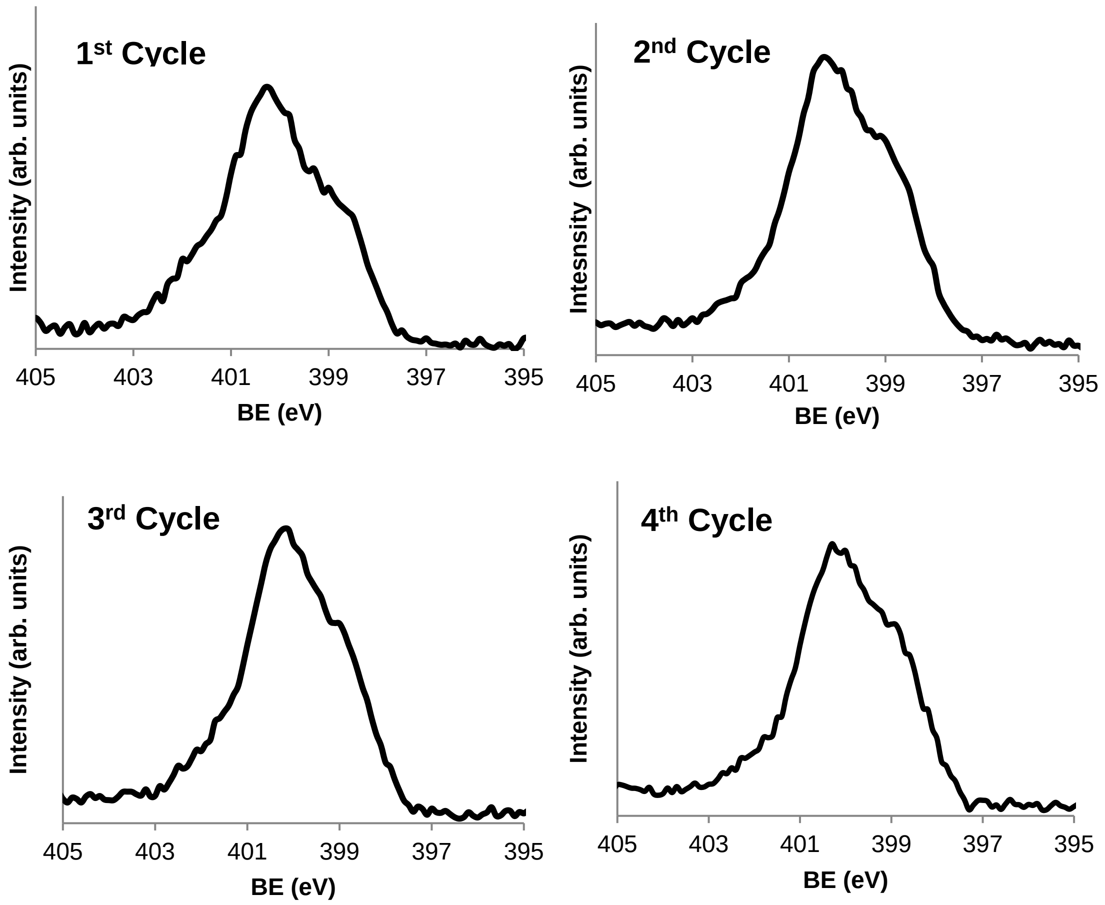

Figure S4. XPS spectra in the N1s region of the re-used G-(H_2L)-Pd catalyst (1-4 cycles).
